# Evaluation of a Mobile Web Application for Assessment Feedback

**DOI:** 10.1007/s10758-021-09575-6

**Published:** 2021-11-10

**Authors:** Mireilla Bikanga Ada

**Affiliations:** The University of Glasgow, Glasgow, UK

**Keywords:** Technology-enhanced assessment feedback, Higher education, Mobile learning, System evaluation, Summative feedback, Formative feedback

## Abstract

This paper reports an evaluation of a mobile web application, “MyFeedBack”, that can deliver both feedback and marks on assignments to students from their lecturer. It enables them to use any device anywhere, any time to check on, and receive their feedback. It keeps the feedback private to the individual student. It enables and successfully fosters dialogue about the feedback between the students and the educator. Feedback and marks were already being delivered using the institution’s learning environment/management system “Moodle”. The study used a sequential explanatory mixed-method approach. Two hundred thirty-nine (239) participants were reported on their experiences of receiving feedback and divided among several groups: (a) feedback delivered in “Moodle”, (b) formative feedback in “MyFeedBack”, and (c) summative feedback in “MyFeedBack”. Overall, results showed a statistically significant more positive attitude towards “MyFeedBack” than “Moodle”, with the summative assessment subgroup being more positive than the formative subgroup. There was an unprecedented increase in communication and feedback dialogue between the lecturer and the students. Qualitative results enriched and complemented the findings. The paper provides guidelines for an enabling technology for assessment feedback. These offer insight into the extent to which any of the new apps and functionalities that have become available since this study might likely be favourably viewed by learners and help achieve the desired pedagogical outcomes. These include: (1) accessible using any device, making feedback accessible anywhere, anytime; (2) display feedback first (before the grade/mark); (3) enable personalisation of group feedback by the teacher; (4) provide privacy for each student; (5) facilitate dialogue and communication about the feedback; and (6) include a monitoring feature. Three goals already put forward in the literature—(1) making the feedback feel more personal, (2) getting a quicker turnround by making it easier for the teachers to achieve this, and (3) prompting more dialogue between the educators and students—are advanced by this study which shows how they can be supported by software, and that when they are achieved then users strongly approve them.

## Introduction

### Feedback

The powerful influence of feedback on the student learning process (Black & Wiliam, 1998; Quality Assurance Agency for Higher Education, 2018; Sadler, 2013; Winstone & Boud, 2020) is widely recognised. Its delivery, which is important to the progress in learning, is a critical component of effective assessment design (Gibbs & Simpson, 2004). There are many perspectives and uses of the term feedback. For example, feedback “*identifies a gap between what is understood/has been demonstrated and the standard of performance expected*” (Price et al., 2010, p. 278). Henderson et al. (2019a) define feedback as ‘*processes where the learner makes sense of performance-relevant information to promote their learning*’ (p. 268). For this paper, which focuses mainly on the vehicle that delivers the feedback rather than the content of feedback, feedback is seen as any type of comment on student assessment. Unfortunately, regardless of the different perspectives or uses of the term, the challenges of feedback in Higher Education are not fading (Dawson et al., 2019; Henderson et al., 2019b). For instance, in the UK National Student Survey (NSS), the assessment and feedback section consistently has lower overall satisfaction scores (MacKay et al., 2019). One of the issues repeatedly reported in the last two decades is the provision of timely feedback in an era of mass participation when universities are struggling with ever-increasing student enrolments coupled with higher demand for assessment (Henderson et al., 2019b).

### Technology-Enhanced Modes of Feedback Delivery

Despite the influence of technology in Higher Education, its effective educational contribution is yet to be fully revealed (Kirkwood & Price, 2014). A growing body of literature agrees that technology is necessary to manage and monitor feedback processes (Deeley, 2018; Nicol, 2009). For instance, the past few years have seen an increase in technology-enhanced modes of feedback delivery such as video, audio, screencast and other annotation feedback mechanisms (Deeley, 2018; Henderson & Phillips, 2014; Mahoney et al., 2018; Wilkie & Liefeith, 2020) and digital tools to facilitate the feedback process (Donia et al., 2018; Pardo et al., 2019). Nevertheless, despite increasing research on feedback, there is no consensus on how the feedback, whether formative or summative, should be designed or delivered. Furthermore, despite some of the technologies such as video and other media being present in the educational sphere for more than twenty years, including the potential use of social software for formative feedback (Hatzipanagos & Warburton, 2009), no much research has been done on technology-enhanced assessment feedback (Henderson & Phillips, 2014, p. 1), particularly, on the use of technology to support feedback production and delivery and student engagement (Hepplestone et al., 2011). In their literature review, Henderson and Phillips found that while substantial literature focused on the feedback itself, few studies investigated the way or medium in which assessment feedback is provided to students (Henderson & Phillips, 2014, p. 3).

Taylor and Burke da Silva (2014, p. 805) suggest looking at whether the feedback delivery mode across schools and disciplines can facilitate more effective feedback. Redecker and Johannessen (2013) argue for a paradigm shift in the use of Information and Communication Technologies (ICTs) in order to support assessment and feedback. On the other hand, despite acknowledging the benefits of technology to deliver timely feedback, Deeley (2018) recommends taking small and incremental steps in the use of technology because it can be challenging and risky; and the mobile platform usage over time and across the yearly cohorts may vary significantly (Stockwell, 2010).

In their study that categorised and analysed research on the educational use of ubiquitous computing, Laru et al. (2015) found that the main challenge was that most tools involved in technology-enhanced learning fields were more concerned with communication and sharing. The potential role of tools and the instructional design that guide and support learning processes were not being highlighted. Indeed, the design, development and delivery of lightweight digital tools and activities for learners are fundamental (Laru et al., 2015).

### Mobile Devices

The potential of mobile learning as a critical element in the transformation of education (Johnson et al., 2014; Traxler, 2010) is still a big topic of discussion. However, the number of studies focused on the perception and adoption of mobile learning is higher than those on its practice (Romero-Rodríguez et al., 2020). Indeed, its use in the educational sector is minimal (Alrasheedi & Capretz, 2013, p. 265). Franklin (2015) remarks that education’s move from the industrial age to the ‘sharing age’ is inevitable in the context of human behaviour and technology (p. 1089). One way the institutions have adopted is to create mobile-optimised versions of their websites or standalone applications that can be downloaded (Chen & Denoyelles, 2013). However, as ownership of the mobile handheld devices, which have spread rapidly and become ubiquitous, has reached the “tipping point” (Franklin, 2011), there is growing pressure for universities to leverage technology that is already in students’ hands, pockets and purses (de Waard, 2014; Phillips et al., 2014). There is a demand for personalisation of the virtual learning spaces students use (Gordon, 2010), and educational institutions are adopting that concept of ‘Bring Your Own Device’ (BYOD) (CISCO, 2012).

Evidence in the literature shows that technology can help manage and monitor the feedback process (Nicol, 2009) and foster dialogue between students and lecturers (Pitt & Winstone, 2020). With the lack of scalability as new trends emerge quickly and the lack of financial support for these ever-changing mobile technologies, it has been suggested to make use of a system that “*allows the leverage of diverse, student-owned technology for academic benefit*” (Ernst et al., 2013, p. 99). Furthermore, there is a continuous demand to adapt learning management systems (LMS) to increase student engagement (Browne et al., 2006; Mensink & King, 2020). However, the difficulty seems to be in finding ‘*readily-available technologies which are quick to learn, easy to use, which are efficient after the start-up period, saving time & effort and increasing productivity and which bring significant learning benefit to students.*” (Ferrell & Sheppard, 2013, p. 4).

However, what is certain is that the current COVID-19 pandemic will have a profound impact on how technology is used for mobile and online learning and assessment feedback.

This research presents the evaluation of prototype technology, “MyFeedBack”, a mobile web application that enables access to assessment feedback using any device and fosters the establishment of communication and feedback dialogue channels between the students and the educator. It also presents the design guidelines for an enabling technology for assessment feedback.

### The “MyFeedBack” Application

**Fig. 1:**
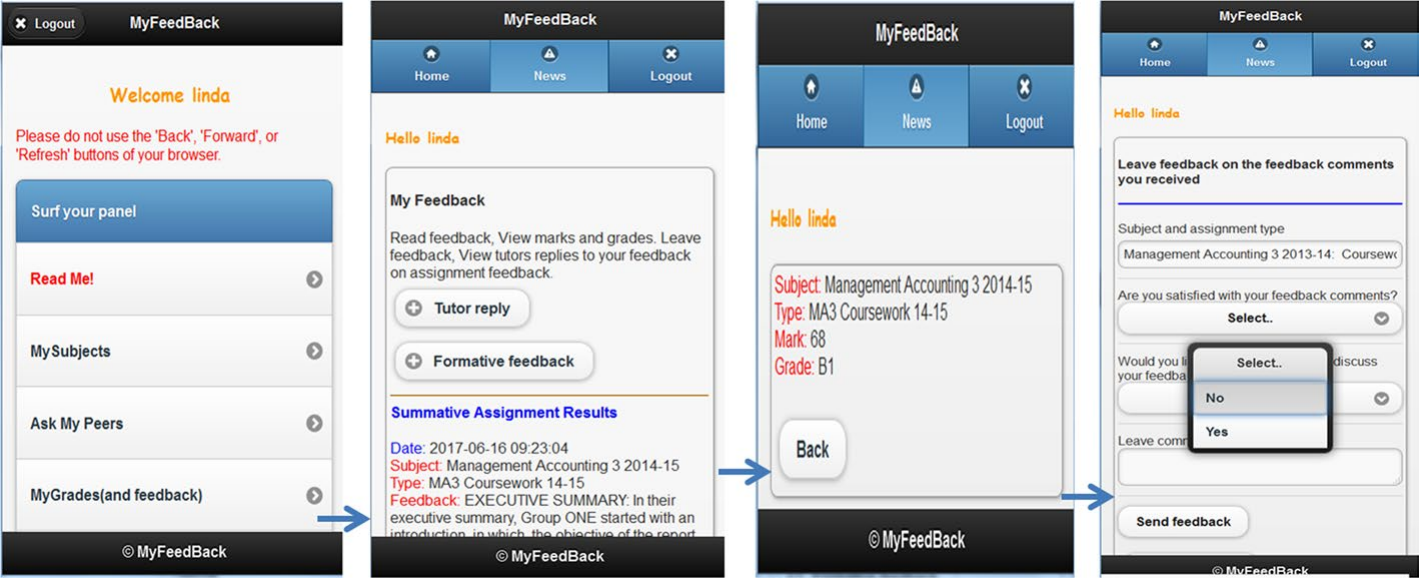
Screenshots of a learner’s interaction with MyGrades

At the time “MyFeedBack” was being developed, the functionality provided by the University’s Moodle was limited and did not meet the requirements of this application. “MyFeedBack” (Bikanga Ada, 2013, 2014a) consisted of five main components for improving communication and feedback dialogue and facilitating access to feedback. These included a discussion board, an assessment feedback feature, a multiple-choice question (MCQ) quiz engine, a peer feedback feature and a polling system. Having different features gave some flexibility to educators who wanted to carry out mobile learning activities with their students. However, the focus of the research was on the assessment feedback feature of the application. While the system allowed uploading individual feedback, it also enabled the lecturers to upload group feedback that they could later modify to reflect individual student contributions to their group assignment. The latter made the process easier and took less time to accomplish. Students needed to log in to use the application and move to the assessment feedback feature (MyGrades), where the first thing they viewed was their summative or formative assessment feedback. In the case of a summative assignment, they could also view their marks. For both types of assignment results, students could use a “Leave Feedback” feature which enabled them to fill in a form to leave comments on their feedback and select ‘yes’ or ‘no’ to the following questions: “Are you satisfied with your feedback comments?”; “ Would you like to meet your lecturer to discuss your feedback?”. The purpose of the feature was to foster communication and feedback dialogue as one of the issues with assessment feedback provision to students is that it is a monologue process, unidirectional from teachers to students (Nicol, 2010). Adding that feature to “MyFeedBack” was an attempt to “close the feedback loop” and “instigate feedback spiral” (Carless and Boud, 2018) while engaging students in the process (Carless, 2015). Figure 1 illustrates screenshots of a student’s interaction with MyGrades.

### Research Questions

The study reported here was part of a study that investigated whether using a mobile web application for assessment feedback increased student motivation, engagement and communication in tertiary education, and through reflection, developed a mobile learning framework for assessment feedback (Bikanga Ada, 2018). Previous work (Bikanga Ada & Stansfield, 2017) had concentrated on students’ behavioural engagement level with their assessment feedback in three studies using digital footprints of students’ access to the ‘MyGrades’ feature. This paper reports on the evaluation of “MyFeedBack”, a mobile web application developed as a means to an end to this research project. “MyFeedBack” enabled access to assessment feedback (formative or summative) using any device and fostered the establishment of feedback dialogue and communication using a feature called “Leave feedback”. This paper also presents the design guidelines for such an enabling technology for assessment feedback.

The research questions are:

RQ1: What are the differences in perceptions towards accessing the assessment feedback on the University Virtual Learning Environment (VLE) Moodle and the “MyFeedBack” Application?

RQ2: Is there any difference in student evaluation of “MyFeedBack” with regards to the type of assessment feedback (for instance, formative vs summative), and can the type of feedback and the type of feedback delivery method increase the level of communication and feedback dialogue?

RQ3: What design guidelines should educational technologists follow in developing systems with characteristics that foster the desired pedagogical outcomes, for instance, increased student behavioural engagement with assessment feedback; increased communication and dialogue about feedback between the educator and students?

## Methods

### Design and Theoretical Paradigm

**Fig. 2:**
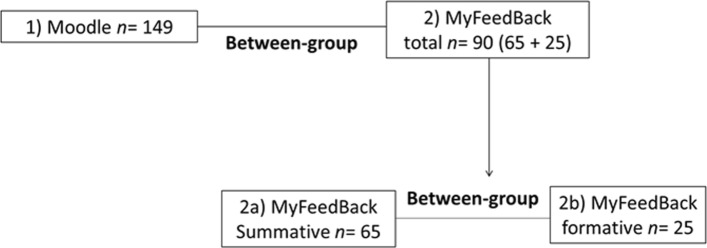
Between-group study design

Mixed methods approaches are increasingly being used to evaluate technology-enhanced learning environments (Mather, 2015). This study uses a mixed-method approach with a pragmatic rationale (Denscombe, 2014). It follows a sequential explanatory design characterised by data collection and analysis of quantitative data followed by qualitative data (Creswell, 2003; Creswell et al., 2003; Creswel & Plano Clark, 2011). An explanatory sequential design enables quantitative components to describe the phenomenon being investigated, while the qualitative elements, based on the subjective experience of participants, bring richness and further meanings to help explain and interpret the quantitative findings or generate new knowledge, resulting in a higher quality of inferences (Creswell et al., 2003; Ivankova et al., 2006; Stange, 2006). The quantitative part followed a between-group design (see Fig. 2) by comparing those results of those who evaluated Moodle against those who evaluated “MyFeedBack”; those involved with the summative assessment feedback with those involved in formative assessment feedback.

### Participants

This study took place in a higher education setting in the UK and focused on assessment feedback. There was a total of 239 students divided into two between-subjects groups, as described in Fig. 2. As shown in Fig. 2, the second group was also divided into two groups: (a) The participants involved in summative assessment feedback (*n* = 65) were from the schools of Health, Nursing and Midwifery (HNM) and Business. Lecturer Amina (not real name) was from the School of Business. Before being introduced to “MyFeedBack”, the lecturer complained of the lack of communication and feedback dialogue despite the extensive feedback she provided over the years for the same module. (b) The second group was concerned with formative assessment feedback (*n* = 25), and the participants were second-year Computing students working on their computing group project. It was not possible to gather the views of their lecturer at the end of the study.

The researcher was not involved in teaching and adhered to Ethical Guidelines for Educational Research (BERA, 2018) by obtaining ethical approval from the University Research Ethics Committee. Participants were informed of the purpose of the study and that any information that could help identify them would not be published. Each participant also signed a consent form.

### Material

The System Evaluation scale was concerned with student attitude to the VLE, Moodle, as a tool for assessment feedback and attitude to “MyFeedBack”. The survey instrument items were adapted from existing studies to ensure the content validity of the scale. These items were adapted from Liaw et al. (2007), Liaw et al. (2008) and Liaw et al. (2010). The reliability reported in those studies, measured by Cronbach’s alpha, was high: alpha = 0.96, alpha = 0.96, and alpha = 0.92, and all the statements were scored on a 7-point Likert scale (ranging from 1, which means “no experience” to 7, which means “highly experienced”). In this study, the statements were scored from 1 “Strongly disagree” to 6 “Strongly agree” with a possible range of 6 – 36. High scores indicated a high level of agreement with the statements. The midpoint was not included in the Likert scales because the study wanted willing participants to provide their true opinions. Research has shown that participants might select the midpoint even if it does not reflect their true opinion or may use it as ‘dumping ground’ (Chyung et al., 2017).

The System Evaluation scale was made of 5 subscales: Learners’ Autonomy which is about students’ ability to take charge of their learning and, in this case, their assessment feedback; System Activities is concerned with the convenience of the tool used; System Satisfaction measures the level of enjoyment in using the tool for assessment feedback; the System functions subscale is concerned with the easiness of use and easiness to retrieve grade and assessment feedback; while the System Acceptance subscale evaluates the level of enhancement on student engagement and motivation with feedback and communication and interaction between the lecturer and the students that the tool can provide. Table 1 presents the System Evaluation scale. The questions were the same and included two open-ended questions; however, the term VLE was replaced with “MyFeedBack” in the second questionnaire. Learner autonomy was coded as “A”, System Activities as “Ap”, System satisfaction as “SA”, System functions as “F” and System Acceptance as “ac”.

**Table 1:** System evaluation questionnaire

F	MyFeedBack is an easy tool for use						
F	MyFeedBack is an easy tool for retrieving my grades						
F	MyFeedBack is an easy tool for retrieving my feedback and reading feedback						
F	MyFeedBack is an easy tool for communicating and interacting with tutor(s)						
SA	I enjoy using MyFeedBack for retrieving grades and reading feedback						
SA	I enjoy using MyFeedBack for communicating and interacting with tutor(s)						
SA	I enjoy using MyFeedBack for sending and retrieving messages						
Ap	MyFeedBack is a convenience tool for retrieving and reading feedback						
Ap	MyFeedBack is a convenience tool for retrieving grades						
Ap	MyFeedBack is a convenience tool for communication and interaction with tutors						
Ap	MyFeedBack is a convenience tool for sending and retrieving messages						
ac	MyFeedBack is a tool for enhancing communication and interaction with tutor(s)						
ac	MyFeedBack is a tool for enhancing engagement with your feedback						
ac	MyFeedBack is a tool for enhancing motivation with feedback						
MyFeedBack mobile application can help me to take charge of my own learning with regards to*:(*1 *means “*Strongly Disagree*” to* 6 *which means “*Strongly Agree*”)*		1	2	3	4	5	6
A	Retrieving grades						
A	Retrieving and reading feedback						
A	Sending and retrieving messages						
A	Communicating and interacting with tutors						

*Q1- Please rate the following statements to evaluate MyFeedBack*

From 1 which means “Strongly Disagree” to 6 which means “Strongly Agree.”

Please only one cross (X) or one tick per line 1 2 3 4 5 6

Q2- Please, list everything you liked about the way you received feedback

Q3- Please, list everything you disliked about the way you received feedback

Beyond the survey, the researcher collected additional data. Student qualitative data came from the open-ended questions in the survey that asked them to list everything that they liked and disliked about “MyFeedBack” (see Table 1 above). Further qualitative data collection was achieved with the online interview questions (see Table 2) using Google form as the researcher was not able to conduct face-to-face student interviews. Qualitative data from the lecturer emerged from her responses to the questions about her experience using “MyFeedBack” for two years. Her opinion was collected using an email interview (Hershberger & Kavanaugh, 2017; Meho, 2006), an appropriate method when the participant is hard to reach. Further data came from observing the interaction between the lecturer and the students on “MyFeedBack” application. Observations allow us to *“determine who interacts with whom, grasp how participants communicate with each other and check for how much time is spent on various activities”* (Kawulich, 2005).

**Table 2:** Online interview questions—students

1	Enter your ID
2	Please tell me what device you used to access your group report feedback on MyFeedBack application and feedback from your other modules. (e.g.: Smartphone, tablet, iPhone, iPad or my PC etc.…)
3	How did you feel about using your mobile handheld device (Smartphone, tablet, iPhone, iPad, etc.…) for mobile learning as complementary to learning e.g.: for feedback? (If you used a PC or Laptop, please also answer the question)
4	What do you believe are the possible barriers to using your own mobile handheld devices (Smartphone, tablet, iPhone, iPad, etc.…) for learning e.g.: for feedback?
5	With MyFeedBack, you can leave your comments on your assessment results immediately using the Leave Comments button so your tutor knows what you think of your results. How do you feel about being able to comment on your assessment results immediately?
6	Please, list everything you believe you liked and everything you disliked about the delivery of MA group report results including feedback using MyFeedBack
7	Comparing feedback delivery methods: How do you feel about the way you received feedback for MA group report (on MyFeedBack) and the way you received feedback from your other modules?
8	How would you feel about accessing your other modules' feedback on MyFeedBack in the future and why?

### Procedure

Participants were asked to evaluate their university’s current technology for delivering their feedback, and those who accessed their feedback through “MyFeedBack” were also asked to evaluate that system. In total, 149 participants had only experienced Moodle at the time they took a survey about Moodle and 90 students who had experienced Moodle in the past but filled in the survey only about “MyFeedBack” after using it.

In the summative assessment group of “MyFeedBack” users, students were asked to access the results for their group reports and (group) presentations, including feedback, which were made available on “MyFeedBack”. For the formative assessment group, student groups were advised to provide a draft of their work every week for five weeks in order to receive formative feedback that would help them towards the final version of their project report. They did not receive any marks or grades.

## Results

A parametric statistical method, Independent-samples t-*test*, was used to analyse the quantitative data using R software. However, where data did not follow a normal distribution and the sample size was small (*n* < 30), a non-parametric statistical method, the Mann–Whitney *U* test, was used (Pallant, 2020). Qualitative data was not extensive and came in different formats, including single word and short answers. Thematic Analysis (Cohen et al., 2017) which allows flexibility in interpreting the data, was used to identify codes or meaning in the participants’ comments, categorise them and finally create the themes where possible without using any specific approach.

### Comparing Virtual Learning Environment Moodle to “MyFeedBack”

The system evaluation involved comparing the university’s current system, Moodle (*n* = 149) to “MyFeedBack” (*n* = 90). An independent-samples t-*test* (Table 3) revealed no significant difference in learner autonomy, System Activities, System Satisfaction and System function scores of the university’s current learning environment (Moodle) and “MyFeedBack” application. However, there was a significant difference in System Acceptance scores of Moodle (*M* = 11, SD = 4.21) and “MyFeedBack” (*M* = 12.4, SD = 4.00; *t*(229) = −2.43, *p* = 0.0156, two-tailed). Results indicated a higher score for “MyFeedBack” app acceptance than Moodle, and a small effect size (Cohen’s *d* = 0.3).

**Table 3:** System differences in measures of learner autonomy, system acceptance, system activities, system functions and system satisfaction

	Moodle			“MyFeedBack”			Mean diff	95% CI	*df*	*t*	*p*	Cohen’s *d*
	*n*	*M*	SD	*n*	*M*	SD						
Learner autonomy	148	16.9	4.57	89	16.6	4.84	.3	− .92, 1.54	235	.498	.6189	.07
System acceptance	149	11	4.21	89	12.4	4.00	− 1.4	− 2.46, − .26	229	− 2.43	.0156	.3
System activities	143	16.6	4.94	90	16.4	4.93	.2	− 1.14, 1.47	231	.246	.805	.03
System functions	139	16.7	4.74	89	16.4	5.01	.3	− .91, 1.68	226	.579	.562	.08
System satisfaction	145	11.5	3.81	89	11.7	3.64	− .2	− 1.19, 0.79	232	− .401	.688	.05

### Is there Any Difference in Student Evaluation of “MyFeedBack” with Regards to the Type of Assessment Feedback, for Instance, Formative vs Summative?

This section focuses on the evaluation of the “MyFeedBack” application with regards to the type of assessment—formative or summative. It involved the “MyFeedBack” users group only, as described in Fig. 2. A Mann–Whitney *U* test (Table 4) revealed no significant difference in Learner autonomy, System Activity, System Satisfaction, and System Acceptance scales. However, there was a significant difference in the scores of System Function scale with a small effect size, of participants involved in summative assessment feedback (*Md* = 18, *n* = 63) and those involved in formative assessment feedback (*Md* = 14, *n* = 25), *U* = 565, *p* = 0.0389, *r* = −0.22. Students in the summative assessment group were more positive about the functions of “MyFeedBack” (higher scores) than those in the formative assessment group.

**Table 4:** Mann–Whitney *U* test results of “MyFeedBack” evaluation based on the type of assessment feedback

	Formative assessment feedback		Summative assessment feedback		*U*	*p*	*r*
	*n*	*Md*	*n*	*Md*			
Learner autonomy	24	16	63	18	639.5	.2676	− .119
System acceptance	25	12	64	12	712	.4221	− .0855
System activities	25	16	65	17	758.5	.6284	− .0515
System functions	25	14	63	18	565	.03891	− .221
System satisfaction	25	12	64	12	702.5	.3738	− .0948

### Communication and Feedback Dialogue on MyFeedBack, for Instance, Formative vs Summative

This section involves participants in the second between-subjects group, as described in Fig. 2 (MyFeedBack – Summative/formative).

#### Summative Feedback on MyFeedBack

**Fig. 3:**
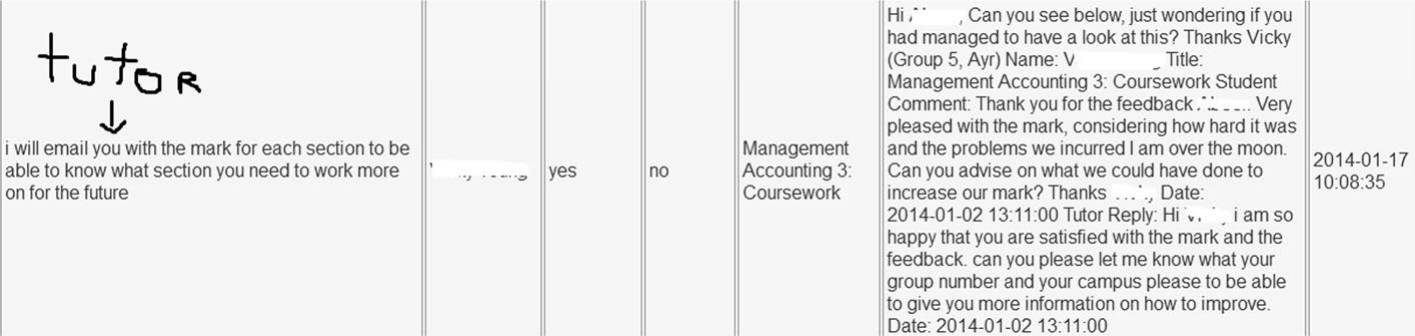
Screenshot of lecturer and student feedback dialogue exchange on “MyFeedBack”

Results emerged from ‘Leave Feedback’, a feature on “MyFeedBack” that enables communication and feedback dialogue. A previous study that looked at student behavioural engagement with assessment feedback using digital footprints showed that a total of 251 students accessed their summative assessment results (Bikanga Ada & Stansfield, 2017). Of these students, 21% used the ‘Leave feedback’ to comment on their assessment results. The lecturers replied to all students’ comments, which included enquiries, meeting requests, an appreciation of their feedback or just some discontentment. In some cases, there was more than one exchange between the students and their lecturer. The feedback dialogue, initiated from the feature ‘Leave Feedback’, continued beyond the “MyFeedBack” application, as seen in Fig. 3. The lecturer also reported an unprecedented increase in email communication about assessment feedback not seen before in all the years she taught that same module. The emails came from students who accessed their feedback.

**Fig. 4:**
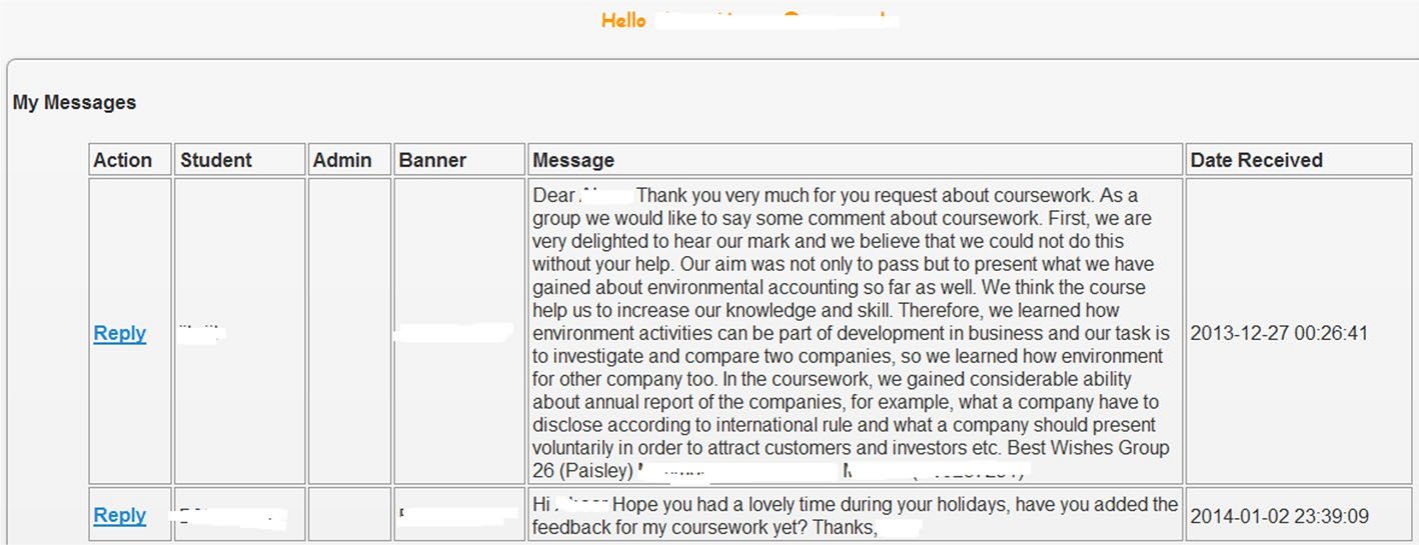
MyMessages feature used by students for communication and feedback dialogue

Some students also made use of another communication feature on “MyFeedBack” to contact their lecturer with regards to their feedback, as illustrated in Fig. 4. The feature used in this case was “MyMessages”, which enables students to leave a message to their tutor. For example, a student who had previously contacted the lecturer regarding the assessment feedback sent a follow-up message. In contrast, another student wrote on behalf of their group and provided an in-depth comment about the coursework.

#### Formative Feedback on MyFeedBack

None of the formative assessment feedback group students commented on their group project formative feedback. Digital footprints of their engagement with their feedback also showed that it was lower than those who received summative assessment feedback (Bikanga Ada & Stansfield, 2017).

### Qualitative Evaluation

#### *What Students Liked and Disliked About “MyFeedBack.”*

The survey also asked students to list everything they liked and disliked about “MyFeedBack”; twenty-six students answered the question. Students found “MyFeedBack”, an easy to use tool that enables anywhere and at any time access while keeping the grade private with individual and personalised feedback: “*It was not available for other persons that is good. You can read it every time again.*” “MyFeedBack” is “*useful and beneficial for feedback”* and “*handy*” for mobiles, and also fosters student engagement with their feedback as the following comment suggests “*Allows comments to be taken on board & developed*”. Some students highlighted the difficulty of using “MyFeedBack” initially as they found the navigation complicated. However, compared to emails, it is the preferred tool for feedback access: “*Don’t like the idea of having to check emails as well as this application but like using it for feedback*.”

#### Student Online Interview Results

Five students from MyFeedBack summative group (see Fig. 2) replied. Four of them used a laptop/notebook, while one used a PC to access their feedback on “MyFeedBack”.

##### Interaction, Communication and Feedback Dialogue

Students were asked how they felt about being able to comment on their assessment results using the ‘Leave feedback’ feature on “MyFeedBack”. One key theme that emerged was communication and feedback dialogue—Having a tool that enables the students to leave comments on their assessment results is ‘good’ as it fosters interaction and feedback dialogue between students and their lecturer. Student A’s comments summed up that feeling:

“I think it is very good because you can put your reply with a query to the lecturer about the results and discuss this further if necessary.” (Student A)

Another aspect is the interactivity and feedback dialogue that leads to further work development, as Student D commented:

“Very much beneficial; allows us to see the lecturer views and opinions on our work to develop” (Student D).

##### Boundaries

Nonetheless, based on her previous experience, Student B is concerned that this may lead to students overwhelming the lecturer with frequent messages. Her views suggest the necessity to separate learning from other activities, implying that the lack of a clear boundary may affect the teaching and learning experience as the real purpose of such a tool becomes lost.

“That’s good. But I believe perhaps for some students it will become a twitting issue, as I have experienced in (campus name removed) campus some student misled the purpose of some facilities.” (Student B).

##### Personalisation

Students were also asked to compare the way they received feedback (on “MyFeedBack”) and the way they received feedback from other modules (these are not on “MyFeedBack”). The personalisation of the feedback is one of the factors that influenced Student A’s opinion of “MyFeedBack”. She thought that their feedback and delivery method were better than her previous experience. She believed that using the same ‘*beneficial*’ feedback delivery method for other modules would be *‘effective and please a lot of students*’ as she commented:

*“The group result was perfect. I have no negative comment about this. It is much better than what we have received in the past, and if all the modules provided this it would be much more effective, and please a lot of students; the “MyFeedBack” [feedback] for the group work was very personalised and very beneficial.”* (Student A).

##### Accessibility, Easiness and Speedy Feedback

Students liked that feedback delivery was fast; the tool was easy to use and feedback accessible. The only dislike was the lack of “MyFeedBack” integration into Moodle (Student D). However, for Student B, although the delivery method was quicker than others and ‘a little bit better’, the feedback itself and the way the module involved in this study is taught were not better.

##### Effective Feedback Delivery Method

All five respondents said they would feel ‘good’ accessing other modules’ feedback on “MyFeedBack” for the same reasons they gave in the previous questions. These include an effective and efficient way of providing personalised, detailed and constructive feedback (Student A and D), anywhere any time access (Student C) and improving both the students and lecturers/tutors (Student B).

“I would feel good because it’s an effective method for receiving detailed and constructive feedback” (Student D)

Furthermore, Student A thought that the issue of *‘generalised feedback’,* which is due to the teacher-student ratio at universities, greatly affects ‘*one-to-one contact*’. For her, the solution lies in using “MyFeedBack”, which is accessible anywhere any time because it is online. She further suggested having it incorporated within all modules.

*“By using this application online, it is easy to access, and I think it would be a great idea to incorporate this within all modules and ask the lecturers/markers to sit down and put a little more effort into our feedback so that we can make use of it.” (Student A).*

#### *Lecturer’s views*

For two years, Lecturer Amina used both her PC and her Smartphone to read her students’ comments on their group report results on “MyFeedBack”. Before being introduced to “MyFeedBack”, the lecturer complained of the lack of communication and feedback dialogue, despite the extensive feedback she always provided for the same module over the years. Her reason for using “MyFeedBack” for two consecutive years rather than using the university’s system or her old way of providing feedback was that “MyFeedBack” enabled the feedback dialogue and communication missing in her teaching. Furthermore, the level of engagement with feedback had increased because of the mobile web application.

Emotions overcame the lecturer as she reported being ‘happy’, which was a marked contrast from an earlier interview, in which she strongly expressed her unhappiness and discontentment due to the lack of feedback dialogue with her students. When asked how she felt about the ‘Leave Feedback’ feature that enabled students to leave a comment immediately after receiving their assessment results and how she felt about reading her students’ comments, her answer reflected that feeling of contentment and fulfilment:

“*I like this feature very much, and I was so happy to have a communication, dialogue with my students on their feedback.”*

## Discussion

This study evaluated “MyFeedBack”, a mobile web application that enables access to assessment feedback (formative or summative) using any device and fosters the establishment of feedback dialogue and communication using a feature called “Leave feedback”. It compared it to Moodle, the university’s virtual learning environment. Results on system evaluation questionnaire items indicated no significant difference in Learner Autonomy, System Activities, System Satisfaction and System functions scores of the university current learning environment (Moodle) and the “MyFeedBack” application. However, there was a significant difference in System Acceptance scores; acceptance scores of “MyFeedBack” were higher than Moodle’s. The system acceptance scale compared both tools on the following themes: enhancing communication and interaction with tutors, enhancing engagement with feedback and enhancing motivation with feedback. Themes emerging from qualitative data supported and enriched these quantitative results.

### Enhancing Communication, Feedback Dialogue and Interaction with Tutors

Results indicated that compared to Moodle, “MyFeedBack” was a tool to enhance communication, feedback dialogue and interaction with their tutors. The critical and easily accessible feature on “MyFeedBack” that enabled students to comment on their feedback and establish a communication and feedback dialogue channel with their lecturer, “Leave Feedback”, could have influenced their views. That feature made it easier and quicker for students to contact their tutors about their feedback with one click as a student commented, “*I think it is very good because you can put your reply with a query to the lecturer about the results and discuss this further if necessary”*. That feature, which “*allows comments to be taken on board & developed*”, was unavailable on Moodle. If students wanted to discuss their feedback, they would have the extra layer of complexity of doing it through email, which many disliked. For many years, the lecturer involved in this research (School of Business—summative assessment feedback) provided the same type of extensively detailed feedback via emails and posted it on the VLE. It was not until “MyFeedBack” that she observed an increased student interaction with feedback, communication and feedback dialogue. The finding confirms that the function of a learning system is important in meeting the needs of students (Huang & Chiu, 2015) and achieve a specific learning objective. The huge demand on lecturers “*to support student access to, and engagement in, feedback exchange*” requires the need to employ appropriate scaffolding tools (Evans, 2013, p. 106) and “MyFeedBack” is one of these tools.

Also, students could have been motivated to engage with feedback and communicate with their tutor because the system enabled private and personalised feedback that *“was not available for other persons*”. As reported in the literature, providing e-feedback via the learning management system could negatively impact students’ willingness to establish feedback dialogue as it seems depersonalised without a way to have a “back and forth” communication (Winstone et al., 2021, p. 637).

### Communication and Feedback Dialogue for Formative Feedback Group

Communication is one of the important affective aspects of learning and assessment and a key challenge for formative assessment (Webb et al., 2018). On the other hand, feedback dialogue has been widely discussed in the literature (Nicol, 2010; Winstone & Boud, 2019). As observed with the summative assessment feedback group, communication and feedback dialogue did not happen in the formative assessment feedback group. It could be that these students saw the whole process as an act of receiving information rather than a process in which they were meant to make sense of and act upon (Henderson et al., 2019a) or seek more clarification in order to improve their group project. Maybe this feedback process was ineffective because students’ feedback literacy (Carless & Boud, 2018) was limited. They may not have known and may not have understood their roles in the process (Henderson et al., 2019a) and may have been used to the monologue and unidirectional process of feedback (Carless, 2015; Nicol, 2010). It could also mean that the formative feedback provided was enough, and given that it was received every week, they did not feel the need to seek further clarifications. The essence of the formative instead of summative feedback could have also influenced this lack of communication and feedback dialogue from the formative feedback group.

### Enhancing Engagement and Motivation with Feedback

Students also thought that “MyFeedBack” was a tool for enhancing engagement and motivation with their feedback as *“you can read it every time again*”, which agrees with the literature that students are more likely to revisit feedback online than written on paper (Parkin et al., 2012). Furthermore, their engagement with “MyFeedBack” could be because it was the feedback they saw first when they logged in and accessed the “MyGrades” feature; they could only access their grade after clicking a button at the end of their feedback which meant they could not avoid seeing their feedback. On Moodle, they could access their grades without seeing their feedback. Literature has reported that when grades are easily accessible, some students do not engage with their feedback (Mensink & King, 2020) or do not read it more than once (Winstone et al., 2021). Additionally, the reason students agreed that “MyFeedBack” was a tool for enhancing motivation could be the following: (1) Motivation is linked to students’ desire to participate in activities (Furió et al., 2015) and “MyFeedBack” allowed access to feedback using any device at a time that was convenient to them, which could have increased their desire for interaction, a critical construct when providing learning activities that improve motivation and control (Frohberg et al., 2009); (2) using their preferred device could have also motivated the students as mobile learning, mobile devices and ownership of devices can lead to an increase in learner motivation (Jones & Issroff, 2007; Metafas & Politi, 2017; Nikou & Economides, 2018; Nitsche, 2013; Sha et al., 2012).

### Accessibility, Easiness, Speedy Feedback and Boundaries

Another aspect that could have influenced student acceptance of “MyFeedBack” over Moodle is its convenience. Indeed, in Winstone et al.’s (2021) study, many students recommended the use of technology that maximises convenience. In this study, the portability and versatility of mobile devices may have considerably encouraged a pedagogical shift from didactic teacher-centred to participatory student-centred learning (Looi et al., 2010), where students are empowered with their own choices, including choices of when and where to access their feedback, what device to use or whether or not to access it in the first place. Furthermore, with the “Leave Feedback” feature, “MyFeedBack” enabled the students to be in control of their choices and their learning (Bikanga Ada, 2018). This is important as there is a demand for adapting LMS to increase student engagement (Browne et al., 2006; Mensink & King, 2020) as “a safe and friendly personal emotional experience environment for learners and improving communication technology” remain some of the “urgent” problems (Liang et al., 2021, p. 174) in leveraging the devices students own, for teaching and learning.

### Design Guidelines for an Enabling System for Assessment Feedback

The development of “MyFeedBack” followed McKenney and Reeves’ (2012) generic model for design research (GMDR) and went through iterative cycles of Analysis/Exploration, Design/Construction, and Evaluation/Reflection phases of GMDR. Modifications were made to the design of “MyFeedBack” in response to the early trials and evaluation. This section proposes the design guidelines educational technologists could follow to develop an enabling technology for assessment feedback and could guide them to establish a design framework that shows the relationship between the pedagogical goals and the interface (Stockwell, 2008). Such a design can have an impact on the system quality, which subsequently can affect learners’ satisfaction and intention to use (Almaiah & Al Mulhem, 2019; Almaiah & Alismaiel, 2019). The guidelines, which may be called principles, requirements, features, aims, goals, functions, or lessons depending on the discipline, emerged from the design process, early trials, evaluation and observations made during the study. These are guidelines for the characteristics a system must have, based on the pedagogic potential that could influence the adoption of technology-enhanced feedback (Pitt & Winstone, 2020), to foster student engagement with assessment feedback and foster communication and dialogue about feedback. They offer insight into the extent to which any of the new apps and functionalities that have become available since this study might be likely to be favourably viewed by learners and could help achieve the desired pedagogical outcomes. However, since results depend not just on the programmer but also on the educators, the learners and the context, the outcomes cannot be guaranteed in an exact way. Therefore, the researcher considers these guidelines as educational effects, not software properties. The researcher’s interpretation of the findings is that in order to achieve the desired pedagogical outcomes, the first six of the seven guidelines must be combined. The seven guidelines for an enabling system for communicating feedback and marks are as follows:

- The system must be accessible using any device
  As observed in this study, some students may not want to use their own mobile handheld devices while others appropriate them for learning. As students carry these devices with them all the time and anywhere, it is critical that students be able to access content using these devices. This gives them the freedom to decide when and where to access it (Fuegen, 2012; O’Bannon & Thomas, 2014; Stockwell, 2010). Students are more motivated when using a system that enables access through a device of their choice. A mobile web application should be considered in order to enable accessibility and widening participation and to limit the possible cost the institution may incur in an attempt to leverage students’ different types of devices.
- The feedback feature of the application must display feedback first (feedback culture change)
  The application was developed to get the students to take notice of and engage with their feedback which was achieved by making sure that the first page they visited was their assignment feedback, and they had to scroll down to click on a button in order to view their grades. In a previous study that looked at behavioural engagement with assessment feedback, digital footprints showed that despite knowing their marks/grades, many students revisited the same feedback several times (Bikanga Ada & Stansfield, 2017), leading us to think that they were engaging with it. Furthermore, grades that are easily accessible may lead to feedback being ignored (Mensink & King, 2020) hence the support for adaptive feedback release (Winstone & Boud, 2020).
- The system must include a feature that enables personalisation of group feedback
  With the use of “MyFeedBack”, participant students were provided with timely, personalised and individual feedback that could be accessed using any device of their choice and anywhere as long as there was internet connectivity. The feature should help reduce teacher workload, one of the main issues highlighted in the literature as lecturers struggle to provide personalised feedback to large cohorts. Personalisation of feedback can increase feedback dialogue (Carless, 2016; Pitt & Winstone, 2018). In this study, group feedback was uploaded first and was later personalised to show individual contributions (Bikanga Ada, 2014b). This also enabled the provision of timely feedback, as uploading individual personalised feedback would have taken more time.
- The system must provide privacy
  Some of the many comments the students left were about privacy. They liked the fact that only they could access and read their own feedback. A login page is, therefore, necessary. Providing privacy implicates a certain level of security, which subsequently has an effect on trust (Almaiah et al., 2019).
- The system must have a feature that facilitates feedback dialogue and communication
  The importance of feedback dialogue and communication has long been stressed in the literature (Carless & Boud, 2018; Nicol, 2010). It is essential that students be able to leave comments on their assessment feedback. The “Leave feedback” feature was incorporated to empower students, giving them a choice to comment and act on the feedback obtained to improve their learning. For example, that feature on “MyFeedBack” asked students whether or not they were satisfied with the feedback provided; whether or not they wanted to meet their lecturer for further feedback discussion and finally, there was a comment box where they could leave their comments on their assessment feedback (Bikanga Ada, 2014a). This study showed that it was possible to establish dialogic feedback channels between the lecturer and students, which subsequently restored the lecturer’s trust in her own assessment feedback practices. Moreover, that feature was introduced to support educators evaluating their own feedback method based on the feedback comments and satisfaction form embedded within that feature.
- The system must include a monitoring feature
  In addition to the assessment feedback features, “MyFeedBack” includes a monitoring tool. It provides information on how many times students access their feedback (Bikanga Ada & Stansfield, 2017; Bikanga Ada, 2014a). Increasingly, monitoring tools such as learning analytics are being used to monitor learner activity (Bikanga Ada & Turinicova, 2020; Hu et al., 2014).
- (Optional) The system must include other features that support other forms of feedback, such as peer feedback
  Even though “MyFeedBack” was used in the context of summative and formative assessment feedback in this study, it can be adapted for other activities (Bikanga Ada, 2014a). For instance, peer feedback, formative assessment in the form of students’ and lecturers’ quizzes, survey and notification features are also included within that application and can suit other teaching and learning styles. For example, the peer feedback feature was added based on the recommendations from a lecturer at conferences at the university. Although these features were not used in the cases studies, they could be used by lecturers when the system becomes integrated with the institution VLE because of any time, anywhere and any device benefits that “MyFeedBack” offers, which implies reaching out to a broader audience.

## Conclusions

This paper presented an evaluation of a prototype mobile web application that enables access to assessment feedback using any devices and fosters the establishment of communication and assessment feedback dialogue channels between the students and the educator. The overall evaluation of “MyFeedBack” was positive. Qualitative data reinforced quantitative results that “MyFeedBack”, a new feedback delivery method suggested by students to be incorporated within all the modules, successfully engaged students with their feedback. Students mostly liked it because it was fast, easy to use and enabled personalised feedback accessible any time and anywhere. It also fostered communication between lecturers and students. The lecturer strongly favoured it because it empowered the students, enabling them to comment on their assessment feedback.

The paper concludes with design guidelines for a system’s characteristics to foster pedagogical outcomes such as student engagement with assessment feedback, communication, and dialogue about feedback between the educators and students. These offer insight into the extent to which any of the new apps and functionalities that have become available since this study might be likely to be favourably viewed by learners and can help achieve the desired pedagogical outcomes. These will enable designers to develop tools to support technology-enhanced assessment feedback in similar ways in other contexts. It will enable researchers to extend their understanding of the requirements of student engagement with their feedback and improving communication and dialogue about feedback. Each guideline may have already been seen in the literature. However, the researcher recommends combining the first six guidelines to achieve the desired pedagogical outcomes.

The important inferences from the findings can be used as a reference for Higher Education institutions to develop assessment feedback delivery medium in an era where the “*identification of low-cost options to support large-scale m-technology integration*” (Koszalka & Ntloedibe‐Kuswani, 2010, p. 141) is crucial as universities are facing significant financial challenges, and yet, they are expected to leverage the use students’ various devices and adopt new ways of teaching. The world of student assessment feedback is complex. Fundamentally, educators need to understand better how students access their feedback and how an enabling technology shapes feedback access while fostering feedback dialogue and communication. A delivery medium that allows flexible access to assessment feedback (any device), personalisation, privacy and enables an easier and faster way to leave comments on the feedback has the potential of improving student engagement with their feedback. However, the study also showed that the ‘Leave feedback’ feature on “MyFeedBack” did not instigate any communication and feedback dialogue in the study involving formative feedback. This is a real area of concern. A continuous effort should be invested in refining the most meaningful and effective feedback and communication dialogue mechanism between the students and the educators. Thus, more research is required to understand better the factors that facilitate the feedback dialogue and communication between the students and their lecturers in a formative assessment context. Research is also required to understand better how the communication and feedback dialogue mechanisms are managed so that student formative assessment feedback is seen as a positive opportunity to improve their work and enhance their learning beyond the time they get that feedback. Educators are also encouraged to reflect on their feedback delivery methods, the feedback they provide, with the aim of understanding how to best instigate or maintain the dialogue with their students.

This study was conducted at one institution. Further studies should include different institutions, modules and be extended to other countries. Although relatively higher, compared to the initial 0% the lecturer had previously experienced, the percentage of students initiating the communication and feedback dialogue channels was lower than 50%. Further studies should seek to increase the sample size, and a mixed-method longitudinal study is required to evaluate the use of a mobile web application for assessment feedback and whether using it for more than one module could improve feedback dialogue and communication between students and lecturers. The study captured qualitative data through free text in questionnaires, email and online google form; additional and rigorous approaches, including individual face-to-face interviews, are needed to capture better aspects of the student experience that can be used to improve communication and feedback dialogue.

This study reinforces three goals already seen in the literature, which are (1) making the feedback feel more personal, (2) getting a quicker turnround by making it easier for the teachers to achieve this, and finally, (3) prompting more dialogue between the educators and students.
